# Clinical and Serological Profile of Myasthenia Gravis in the O’Higgins Region of Chile: A Regional Study

**DOI:** 10.7759/cureus.88829

**Published:** 2025-07-26

**Authors:** Manuel Orellana, Miguel González, José Muñoz, Felipe Maragaño

**Affiliations:** 1 Neurology, Dr. Franco Ravera Zunino Hospital, Rancagua, CHL; 2 Medicine, University of O'Higgins, Rancagua, CHL

**Keywords:** anti-achr antibody, autoimmune neuromuscular disease, current treatments, health public, hospital epidemiology, local prevalence, myasthenia gravis (mg), ravulizumab

## Abstract

Myasthenia gravis (MG) is a rare, chronic, and progressive autoimmune disorder that can lead to significant disability in its advanced stages. This retrospective observational study aimed to estimate the prevalence of MG in the O’Higgins Region of Chile and provide a comprehensive characterization of patients’ clinical profiles, including medical history, diagnostic findings, and therapeutic responses. A female predominance (n=47, 66.2%) was observed, and most patients showed abnormalities in electrophysiological studies. In nearly all cases, diagnosis was established through autoantibody testing, with acetylcholine receptor antibodies being the most frequently identified. The most frequent comorbidities included mood disorders, hypertension, and thyroid dysfunction. Most patients received cholinesterase inhibitors; corticosteroids were used in 74.6% of cases, and one-third were treated with conventional immunosuppressants. A smaller group received biological therapies such as rituximab or other newer agents, ravulizumab. Despite the range of available treatments, approximately 20% of patients exhibited a refractory disease course. MG is a disease with a multifaceted impact on patients' personal, social, and occupational lives. The findings of this study may support the development of targeted health policies aimed at improving comprehensive care for MG patients in Chile.

## Introduction

Myasthenia gravis (MG) is an autoimmune disease caused by the presence of antibodies that specifically bind to various postsynaptic components of the neuromuscular junction, thereby disrupting neuron-to-muscle communication [1]. The electrical impulse (as an action potential) originating from the nervous system is altered in its transmission to the muscle fibers. Clinically, MG is therefore characterized by the presence of fatigable muscle weakness, which may present with different patterns throughout the course of the disease [2]. Due to its autoimmune nature, the thymus plays a predominant role in the pathogenesis of this condition, with thymic hyperplasia or thymoma observed in 65% or 10-15% of patients, respectively [1]. 

Given its heterogeneous presentation, diagnosis requires a clinical evaluation of symptoms and signs-particularly demonstrating muscle fatigability-followed by confirmation through the detection of autoantibodies and/or an electromyographic study. Currently, the management of MG involves pharmacological treatment primarily with anticholinesterase agents and various types of immunosuppressants, as well as surgical treatment (thymectomy) [3]. 

Although MG currently has no definitive cure, pharmacological and surgical treatments can reduce symptoms and prevent hospitalizations in the majority of cases. Despite the favorable outcomes of current therapies, there is a smaller proportion of refractory patients whose symptoms may affect their social and occupational lives [4]. 

The estimated prevalence of MG varies widely across different continents. MG prevalence estimations range from one to 30 patients per 100,000 inhabitants [5]. MG is more frequent in women than in men, with an estimated ratio of 2:1 [5]. In Chile, in 2018, the prevalence of MG was estimated at 8.3 patients per 100,000 inhabitants using an indirect capture-recapture method, an indirect method of estimating population sizes [6-8]. 

The estimation of the prevalence of MG as a rare neuromuscular disease allows for improvements in health policies for these patients, allocating the necessary resources and more efficiently planning the rehabilitation and comprehensive support needs that these patients require [9]. 

To contribute with scientific information to the improvement of health policies, the present study seeks to estimate the prevalence of MG in patients treated within the public health system across the entire O’Higgins Region of Chile, along with a comprehensive description of the status of these patients, including clinical history, diagnosis, comorbidities, hospitalizations, symptoms, and response to current pharmacological treatments. 

## Materials and methods

Study design 

This is a retrospective observational study. Patients were initially assessed at their local Centro de Salud Familiar (CESFAM), of which there are 32 across the O’Higgins Region of Chile. Based on clinical suspicion, they were referred to lower-complexity regional node hospitals equipped with neurology services, including Santa Cruz Colchagua Hospital (Santa Cruz), San Juan de Dios Hospital (San Fernando), and Ricardo Valenzuela Sáez Hospital (Rengo). Following this secondary evaluation, patients were further referred to the Dr. Franco Ravera Zunino Regional Hospital in Rancagua, Chile, the region’s reference center, where specialized neuroimmunology assessments were performed to confirm the diagnosis and initiate or optimize treatment.

In accordance with the Good Clinical Practice of the International Council for Harmonization guidelines (GCP-ICH) regarding the protection and confidentiality of patient data, the present study was approved by the Hospital Ethics Committee of Dr. Franco Ravera Zunino Hospital (approval number: EI-FRZ-2025-017), where the research was conducted. 

Patient selection

Patients presenting clinical manifestations of the disease along with positive results in any of the confirmatory diagnostic tests described were included in the registry. No patients were excluded based on age or MG manifestation type. Patients with suspected MG but without complete records were not included.

Data collection, storage, and analysis 

Data extracted from each patient’s medical record included: anonymized patient identification code, age, sex, electromyography results, immunological test results, antecedent pharmacological treatment, number of hospitalizations in the past year, history of thymectomy, current pharmacological treatment, presence of persistent symptoms despite treatment (refractory MG), adverse drug reactions, and comorbidities. 

The anonymized patient data were stored in a password-protected Microsoft Excel spreadsheet (Microsoft Corporation, Redmond, Washington, United States) on a computer with restricted access, available only to members of the research team. Data analysis was performed within the same file, and only the results were exported for the subsequent drafting of the manuscript. 

Diagnosis of MG 

The diagnosis is initially based on the evaluation of characteristic signs and symptoms of MG, particularly demonstrating muscle fatigability, alongside serological testing for autoantibodies and electrophysiological studies. Repetitive nerve stimulation testing and single-fiber electromyography allow for the identification of neuromuscular junction abnormalities. These tests are recommended in patients with negative serological tests for anti-acetylcholine receptor antibodies (anti-AChR) and anti-muscle-specific tyrosine kinase antibodies (anti-MuSK). 

Electromyography 

Repetitive nerve stimulation test: Patients suspend treatment with pyridostigmine for at least six hours prior to the test. Briefly, trains of six square-wave pulses are applied through bipolar stimulation electrodes at a frequency of 3 Hz, and surface recording electrodes are placed such that the initial nerve stimulation elicits a compound muscle action potential. Muscle selection is based on clinical presentation. An abnormal test result is defined as the ratio between the first and the fourth or fifth amplitude of the wave. A decrease of more than 10% (of wave amplitude) in at least one muscle is considered a pathological result [9]. 

Single-fiber study: This technique compares the action potentials of two muscle fibers innervated by the same motor axon. The conduction delay between one and the other is referred to as "jitter." The study is considered abnormal if the mean jitter value exceeds the upper limit of the normal value or if more than 10% of the pairs show increased jitter (more than two of 20 pairs) [10,11]. 

Detection of Plasma Anti-AChR and Anti-MuSK Autoantibodies

Using the radioimmunoassay (RIA) technique, anti-acetylcholine receptor antibodies (anti-AChR) and anti-muscle-specific tyrosine kinase antibodies (anti-MuSK) are detected as biomarkers. A result ≥ 0.02 nmol/L for either antibody is considered positive [12,13]. 

Statistical analysis 

A population pyramid was constructed by grouping patients into 10-year age intervals and calculating the absolute and relative frequency within each age group. Comparative analyses by sex were performed using chi-square tests for categorical variables and t-tests or non-parametric equivalents (e.g., Mann-Whitney U test) for continuous variables, as appropriate. In the diagnosis and treatment sections, variables were categorized, and the frequency of each was recorded in contingency tables. Proportional distributions were calculated for each categorical variable using the total patient cohort as the denominator, unless otherwise specified. 

## Results

Regional prevalence of MG

The specialty outpatient clinic of the Regional Hospital had a registry of 71 patients diagnosed with MG. According to the national census conducted in 2024, the O’Higgins Region has a population of 987,228 inhabitants; therefore, the estimated prevalence is 7.2 individuals per 100,000 inhabitants [14]. 

**Figure 1:**
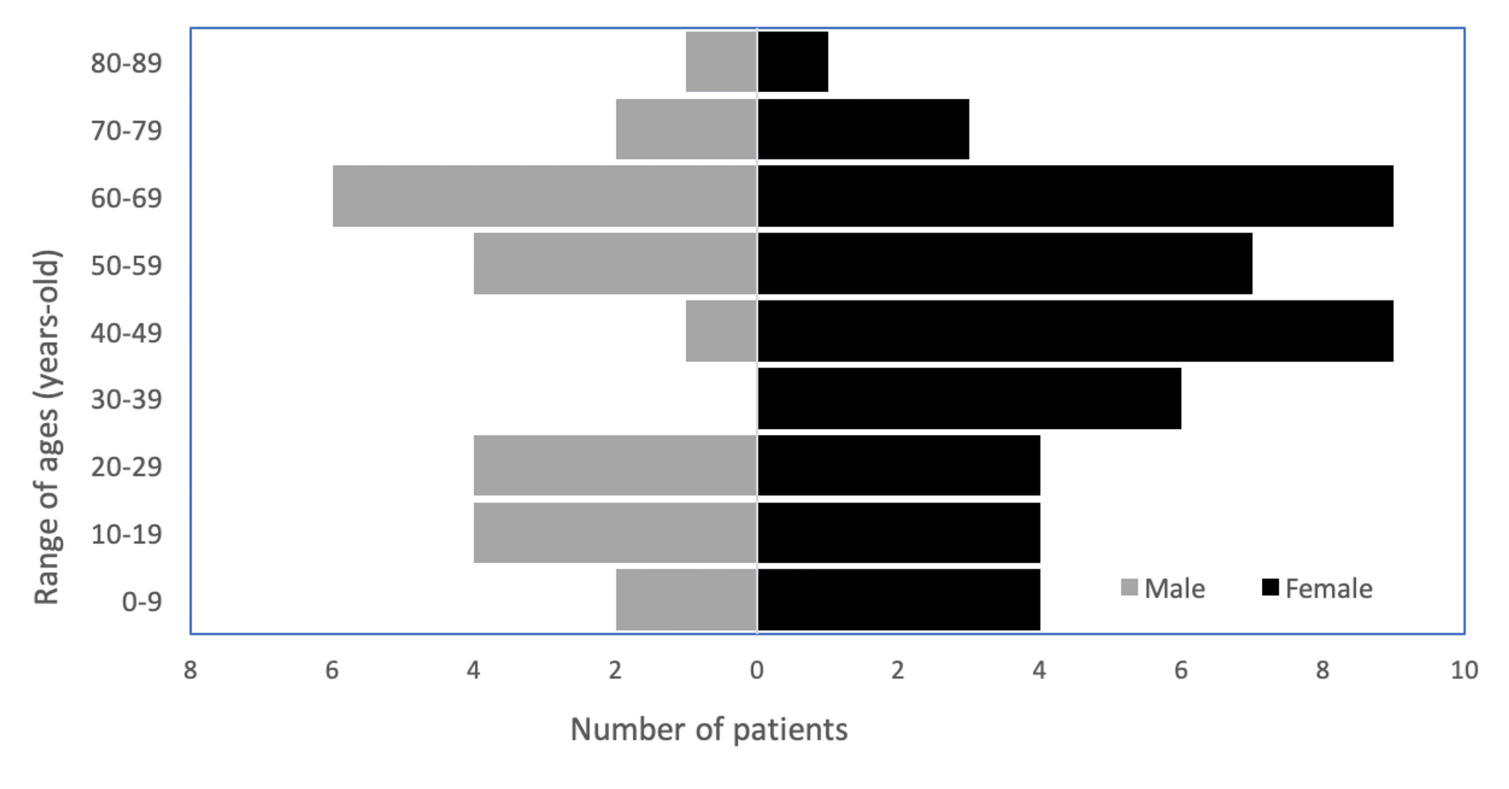
Population pyramid of myasthenia gravis patients.

Of the total number of patients, 24 (33.8%) were men and the remaining 47 (66.2%) were women. When analyzing the age distribution of the population, a higher number of women was observed in the age group of 30-69 years. A complete description of the population is detailed in Figure 1. 

The registry included a wide age range at diagnosis, spanning from 1.5 years (infant) to 86 years (elderly adult). Consequently, the time since diagnosis also varies considerably among patients, with a median disease duration of 6.5 years. 

Diagnosis 

Among the registered patients (24 men and 47 women), 56 (78.9%) exhibited abnormalities in either repetitive nerve stimulation or single-fiber electromyography. This proportion was slightly higher in women than in men (78.7% (37/47) vs. 79.2% (19/24), respectively) (Table 1). 

**Table 1:** Distribution of diagnostic outcomes across sexes and test types A total of 71 patients underwent electromyography, and 68 underwent radioimmunoassay. ND: not determined; RIA: radioimmunoassay; AChR: acetylcholine receptor antibody; MuSK: muscle-specific kinase antibody

Method of diagnosis	Male			Female			Total
	Positive, n (%)	Negative, n (%)	ND, n (%)	Positive, n (%)	Negative, n (%)	ND, n (%)	Positive, n (%)
Altered electromyography	19 (79.2)	5 (20.8)		37 (78.7)	10 (21.3)		56 (78.9)
RIA Anti-MuSK	0 (0)	15 (62.5)	9 (37.5)	1 (2.3)	31 (70.5)	12 (27.3)	1 (1.5)
RIA Anti-AChR	23 (95.8)	1 (4.2)		33 (75)	11 (25)		56 (82.4)

Serological testing for anti-acetylcholine receptor antibodies (anti-AChR) was positive in 23 of 24 men (95.8%) and in 33 of 44 women (75%). Overall, 56 of 68 patients (82.4%) tested positive for anti-AChR. In contrast, only one woman tested positive for anti-MuSK antibodies, representing 1.5% of the total cohort. Serological testing for autoantibodies (anti-AChR and/or anti-MuSK) was performed in 68 of 71 patients (95.8%) (Table 1). A total of 11 patients (16.2%) had negative serology for both anti-AChR and anti-MuSK. This is the first study in Chile where RIA tests were used to confirm the diagnosis of MG in compliance with international guidelines.

Comorbidities 

The main comorbidity identified in the registry was depression and/or generalized anxiety disorder, present in 18 (25.4%) patients. Arterial hypertension was more frequent in men (n=8; 33.3%) than in women (n=9; 19.1%). Conversely, a higher prevalence of thyroid disorders was observed in women (n=13; 27.7%) compared to men (n=3; 12.5%). As a consequence of MG progression, refractory palpebral ptosis was more frequently seen in women (n=13; 27.7%) than in men (n=2; 8.3%). Conditions reported with a frequency below 10% included: type 2 diabetes (n=6; 8.5%), fibromyalgia (n=2; 2.8%), and rheumatoid arthritis (n=2; 2.8%) (Table 2). 

**Table 2:** Comorbidities registered in patients with myasthenia gravis

Comorbidities	Male, n (%)	Female, n (%)	Total, n (%)
Depression and/or anxiety	5 (19.2)	13 (27.6)	18 (25.4)
Arterial hypertension	8 (33.3)	9 (19.1)	17 (23.9)
Thyroid disorders	3 (12.5)	13 (27.7)	16 (22.5)
Refractory eyelid ptosis	2 (8.3)	13 (27.7)	15 (21.1)
Type II diabetes	3 (12.5)	3 (6.4)	6 (8.5)
Fibromyalgia		2 (4.3)	2 (2.8)
Rheumatoid arthritis	1 (4.2)	1 (2.1)	2 (2.8)

Pharmacological and surgical treatments 

The first-line pharmacological treatment for MG is pyridostigmine (acetylcholinesterase inhibitor); indeed, 68 (95.8%) patients receive this drug as therapy. For patients who experience adverse drug reactions to the generic formulation of pyridostigmine, the innovative formulation (Mestinon®) was offered. Pyridostigmine treatment is frequently used in combination with a corticosteroid such as prednisone, which was the second most commonly used drug in this registry for 52 patients with MG (73.2%). The most frequently prescribed doses were 5 and 10 mg per day (46.5%). If disabling symptoms could not be controlled, a non-steroidal immunosuppressant, such as azathioprine, was added to the therapy. A total of 22 (31%) patients in the registry receive this medication. In those who experienced an adverse reaction to azathioprine (9.8%), it was replaced with mycophenolate (n=9; 12.6%). If symptoms persisted despite this drug combination, rituximab or ravulizumab was added (14; 19.7% or 1;1.4%, respectively) (Table 3).

**Table 3:** Surgical and pharmacological treatments

Treatments	Male, n (%)	Female, n (%)	Total, n (%)
Thymectomy	5 (20.8)	12 (25.5)	17 (23.9)
Acetylcholinesterase inhibitors			
Pyridostigmine	24 (100)	44 (93.6)	68 (95.8)
Corticosteroids			
Prednisone	18 (75)	34 (72.3)	52 (73.2)
Deflazacort		1 (2.1)	1 (1.4)
Immunosuppressants			
Azathioprine	8 (33.3)	14 (29.8)	22 (31)
Mycophenolate	3 (12.5)	6 (12.7)	9 (12.6)
Anti-CD20 antibody			
Rituximab	1 (4.2)	13 (27.7)	14 (19.7)
Complement inhibitors			
Ravulizumab		1 (2.1)	1 (1.4)

As shown in Table 3, 17 patients (23.9%) underwent thymectomy. Of these, the majority (94.1%) were under 65 years old at the time of surgery. Despite the described treatment options, 15 patients (21.1%) were found to have refractory MG, with a markedly higher prevalence in women (n=14; 29.8%) compared to men (n=1; 4.2%). 

To enhance the follow-up description of these 71 patients under treatment, hospitalizations due to symptom exacerbation during the 12 months preceding data collection were recorded. It was seen that a total of 13 patients (18.3%) were hospitalized due to symptom exacerbation in the past year, of which six patients (46.1%) had refractory MG.

## Discussion

In this study, we characterized the population of patients with MG treated at the Dr. Franco Ravera Zunino Regional Hospital in the O’Higgins Region of Chile, which accounts for 5.3% of the national population [14]. The estimated regional prevalence of MG was 7.2 per 100,000 inhabitants, a figure consistent with the recent findings of Cea et al. (8.3 per 100,000 inhabitants) [6]. In contrast, Argentina, a neighboring country, has reported a considerably higher prevalence (36.7 per 100,000 inhabitants), underscoring the variability in MG prevalence across different populations and geographic regions [15].

Chile and other Latin American countries lack national epidemiological studies on MG; however, our regional study is consistent with other parts of Latin America, where prevalence figures are generally lower than 10 per 100,000 population. This contrasts with records from the Iberian Peninsula, where prevalence is higher [16]. For example, Colombia’s National Registry of Rare Diseases estimated a prevalence of 1.78 per 100,000 inhabitants in 2015 [17], while the only available study from Uruguay in 1975 reported a prevalence of 6.3 per 100,000 inhabitants [18].

Previous epidemiological studies show that MG is more prevalent in women (2:1) [5]. In our cohort, we observed a higher female predominance, with a ratio of 1.96:1 (47 females to 24 males), especially between the ages of 20 and 69 years. This compares with the reported predominance of early-onset MG in women younger than 50 years [16]; moreover, a recent French population-based study reported a gradual increase in the incidence of MG from the age of 40 years in women and from the age of 60 years in men [19].

Our findings confirm that the majority of patients in our cohort tested positive for anti-AChR antibodies (82.4%), consistent with the serological profile commonly associated with generalized MG [5]. This proportion is in line with previous studies reporting anti-AChR antibody positivity in approximately 80-85% of cases [20]. In contrast, only one patient (1.5%) tested positive for anti-MuSK antibodies, a prevalence notably lower than the 5-8% typically reported in the literature [21]. Notably, 11 patients (16.2%) were double seronegative, with no detectable antibodies against either AChR or MuSK. This subgroup presents a diagnostic challenge, as these patients may be underdiagnosed; therefore, current diagnostic algorithms recommend repeating antibody testing or exploring alternative antibodies such as anti-LRP4 (Low-Density Lipoprotein Receptor-Related Protein 4) or agrin [22,23].

In a cross-sectional study of patients affiliated with the German Myasthenia Association, it was observed that 31% had symptoms of depression and anxiety disorders, also affecting the mental health of caregivers of patients with MG [24]. This comorbidity was the most frequent in our registry, underscoring the importance of complementary therapies to support these patients. The high frequency of hypertension may be attributed to a combination of factors: poor healthy lifestyle habits, an adverse effect of prednisone, and alterations in the cardiovascular physiological mechanisms involved in blood pressure regulation, which are inherent to the nature of the disease [25]. Patients with MG have a higher risk of developing thyroid disorders, which is consistent with the findings in our registry, where it is one of the most frequent comorbidities [26]. 

Although pharmacological treatment is based on international clinical guidelines, the heterogeneity of clinical manifestations, persistence of symptoms, intolerance to medications, and variability in treatment response result in diverse pharmacotherapy combinations [27]. Regarding immunotherapy, in line with international experience [3] as well as a case reported in Chile [28], nearly 20% of patients in our registry receive rituximab as part of their treatment. Ravulizumab is among the newer treatment options [29]; one patient with refractory MG received this immunotherapy, resulting in a marked improvement in symptom control. 

Approximately 21% of patients do not respond to conventional treatment or present some form of intolerance, resulting in refractory myasthenia gravis (refractory MG), a condition defined by persistent clinical symptoms or exacerbations despite adequate use of standard therapies, such as cholinesterase inhibitors, corticosteroids, and at least one immunosuppressive agent [30]. This finding is consistent with a previously published article, which reported that despite the variety of available treatments, a significant proportion of patients experience symptoms that impact their family planning, social life, and occupational performance [31]. 

Limitations

This study has some limitations that are inherent to the retrospective observational design, as all collected data depend on the accuracy of information in existing clinical registries. This may lead to information bias, mainly in variables such as mild adverse effects and/or comorbidities. It should be noted that although the total number of patients diagnosed in the public network of this region was included, the sample size is small (n=71). In addition, there is a possibility of under-reporting of cases, for example, those patients treated in the private health system who were not referred to public health centres, leading to an underestimation of the prevalence reported above. These results could not be directly extrapolated to other regions of the country, given that the geographical representativeness is restricted to a specific area with particular health characteristics; however, it corresponds to the basis of new prevalence searches in other regions and even in the country. Another relevant limitation was the absence of functionality scales such as Myasthenia Gravis Activities of Daily Living (MG-ADL) or Quantitative Myasthenia Gravis (QMG), which restricts the clinical and therapeutic impact on the population studied. This opens the way for future studies to consider these scales. It should also be noted that a small number of patients did not have complete serological and electromyographic results.

## Conclusions

This descriptive study of MG addresses a low-prevalence but clinically significant condition that profoundly affects patients due to its chronic and disabling nature. The O’Higgins Region, being a relatively small and less densely populated area compared to the Metropolitan Region, allowed for effective coordination and data collection, contributing to the regional representativeness of the sample. This study represents not only a pilot effort in characterizing the disease locally but also a potential foundation for developing and implementing comprehensive support strategies for patients and their families.
